# Nurses’ Perception of Safety Culture in Medical−Surgical Units in Hospitals in Saudi Arabia

**DOI:** 10.3390/medicina58070897

**Published:** 2022-07-04

**Authors:** Bader A. Alrasheadi, Majed S. Alamri, Khalid A. Aljohani, Reem AL-Dossary, Hamdan Albaqawi, Jalal Alharbi, Khaled Al Hosis, Mohammed S. Aljohani, Noura Almadani, Rawaih Falatah, Jazi S. Alotaibi, Joseph U. Almazan

**Affiliations:** 1 Nursing Department, College of Applied Medical Sciences, Majmaah University, Majmaah 11952, Saudi Arabia; b.alrasheadi@mu.edu.sa (B.A.A.); jalotaibi@mu.edu.sa (J.S.A.); 2 Nursing Department, College of Applied Medical Sciences, University of Hafr Al Batin, Hafr Al Batin 39524, Saudi Arabia; jalaln@uhb.edu.sa; 3 Community Health Nursing Department, Nursing College Taibah University, Al-Madinah 42351, Saudi Arabia; kajohani@taibahu.edu.sa; 4 Nursing Education Department, Nursing College, Imam Abdulrahman Bin Faisal University, Dammam 34212, Saudi Arabia; rnaldosari@iau.edu.sa; 5 College of Nursing, University of Hail, Hail 55476, Saudi Arabia; h.albaqawi@uoh.edu.sa; 6 Department of Nursing Education, Nursing College, Qassim University, Buraydah 52571, Saudi Arabia; 3747@qu.edu.sa; 7 Medical and Surgical Nursing Department, Nursing College, Taibah University, Al-Madinah 42351, Saudi Arabia; msejohani@taibahu.edu.sa; 8 Community Health Nursing Department, Nursing College, Princess Nourah bint Abdulrahman University, Riyadh 11564, Saudi Arabia; naalmadani@pnu.edu.sa; 9 Nursing Administration & Education Department, College of Nursing, King Saud University, Riyadh 12371, Saudi Arabia; rfalatah@ksu.edu.sa; 10 Nursing Department, Nazarbayev University School of Medicine, Nursultan 020000, Kazakhstan; joalmazan030@gmail.com or

**Keywords:** health care, patient safety, patient care, medical errors

## Abstract

*Introduction:* Patient safety captures the essence of the primary principle of medical ethics, primum non nocere, first do no harm; this is an important concern in the health care system. Nurses are indispensable members of this system and are the largest group of health care providers involved in the direct delivery of patient care. As an integral part of the health care system, it is important to know nurses’ opinions on patient safety culture. *Objectives:* First, to evaluate and measure the existing safety culture and safety of patients in medical−surgical wards (MSW) in hospitals located in the Qassim region, in the Kingdom of Saudi Arabia. Second, to survey the opinion of registered nurses and supervisors/managers about safety culture and issues concerned with safety in hospitals in the region. *Materials and Methods*: A validated cross-sectional survey, namely the Hospital Survey on Patient Safety Culture (HSOPSC), was used. This survey queried 300 nurses in different MSWs in four hospitals in the Qassim Region. *Results:* Overall, a positive culture of safety exists in MSWs, with 69% of RNs rating their wards as having great/excellent safety culture. Notably, some participants felt it was problematic that blame was assigned to nurses for reported errors. While 55.9% of participants noted that all errors or narrowly avoided errors had been reported, less than half actually reported errors in the last year. *Conclusion:* The perceived safety culture was largely positive; however, the results also indicated that a culture of safety comes with some risk and blame.

## 1. Introduction

According to the World Health Organization (WHO), patient safety includes delivering health care that minimizes risks and harm to patients, including avoiding preventable injuries and reducing medical errors [1].

An organizational and multidisciplinary approach, in which nurses play the fundamental role, is essential for patient safety. A culture of safety includes characteristics such as frame of mind, perceptions, notions, beliefs, and values with regard to the safety of the patient [2].

Patient safety culture has an impact on staff and patient health outcomes [3,4]. Many types of safety cultures exist in the workplace—reactive, proactive, positive, negative, etc. A positive culture is considered very important for the organization as it reflects the quality and effectiveness of its communication, an important tool in order to achieve patient safety goals and to prevent incidents. Communication across the organization is considered a key indicator of patient safety culture reflecting reciprocal trust, flow of information, individual perceptions regarding the importance of patient safety, institutional learning, leadership, the organization’s commitment, and the existence of an exonerating approach to incident and error reporting [5]. Nurses, the largest group of direct health care providers, are indispensable members of the health care system; therefore, they shoulder the responsibility of patient safety in hospitals. Just the implication of a medical error impacts the overall health care system, including nursing professionals. Measuring the existing safety culture of hospitals and the perceptions of nurses toward a safety culture enables the identification of strengths, areas for improvement, and possible interventions. This study aimed, first, to evaluate and measure the existing safety culture and safety of patients in medical−surgical wards (MSW) in hospitals located in the Qassim region, in the Kingdom of Saudi Arabia. Second, it aimed to survey the opinion of registered nurses and supervisors/managers about safety culture and issues concerned with safety in hospitals in the region. 

## 2. Methodology

A validated cross-sectional survey, the Hospital Survey on Patient Safety Culture (HSOPSC) from the Agency for Healthcare Research Quality, was used [6]. The survey was conducted in the medical−surgical wards (MSW) of four hospitals in the Qassim region, Kingdom of Saudi Arabia (KSA). Using the universal sampling method, all of the qualified nursing staff in the wards were included in the research.

HSOPSC was disseminated to 300 nurses in 16 MSW in the four hospitals in Qassim, KSA. Two hundred and eighteen (*n* = 218) nurses completed the survey, a response rate of 73%. HSOPSC was developed by Westat Rockville and colleagues at the Agency for Healthcare Research and Quality, Department of Health and Human Services, USA. Psychometric validation measures were found to be adequate based on reliability and factor analysis outcome [6]. The original first version of the tool was used. It has various sections, but the portion measuring perception related feedback has 18 Likert scale items scored on a 1 (strongly disagree) to 5 (strongly agree) range [6].

The inclusion criteria were nurses registered with the Saudi Commission for Health Sciences and working in medical and surgical wards of the target hospitals. The exclusion criteria were nurses working in non-surgical wards such as those posted in pediatric, psychology, and ICU, and those without a valid registration with the Saudi Commission for Health Sciences, such as nursing assistants and nursing interns.

The number and the percentage of positive-worded items and negative-worded items from the survey were calculated. This analysis is in line with recommendations of the AHRQ survey (2004) [6]. The collected data were analyzed with descriptive and qualitative statistics using the SPSS version 22.0 and all of the original output tables/graphs are presented.

## 3. Result

Table 1 details the location, numbers, and percentages of survey participants.

**Figure 1:**
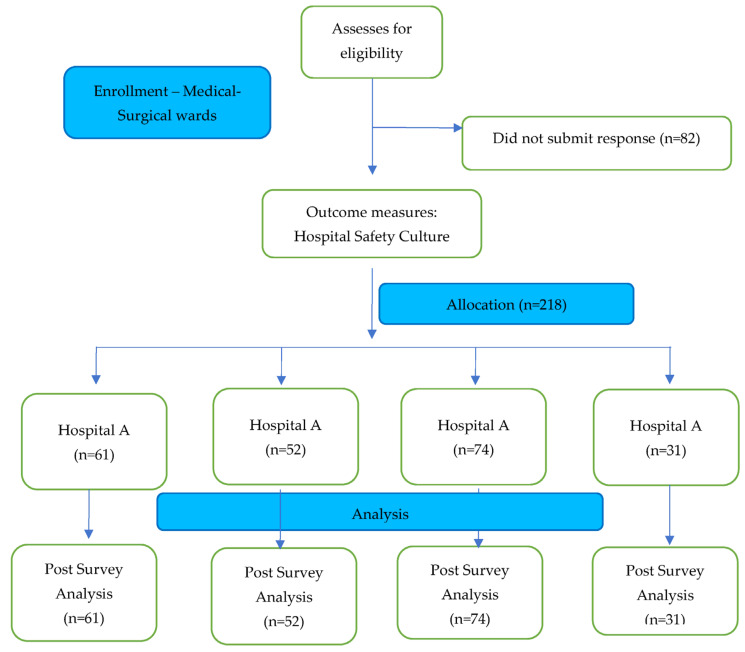
Flowchart representation of the study.

Figure 1 provides a flowchart of the study responses: 300 subjects were assessed for eligibility and 218 subjects completed the survey.

### 3.1. Background Information and Respondent Details

This section provides background information on the nurse respondents, such as their position in the hospital and their working experience. Approximately one quarter of study participants had worked in the same hospital for 6 to 10 years, while the majority had been working for less than 5 years, and their working hours were between 40 and 59 h per week (the average work week in Saudi Arabia is 40 h). The majority (82%) of participants were registered nurses (RN) at the bedside, and 17% were either charge nurses or nurse supervisors. 

### 3.2. Patient Safety Grade 

Overall, the results indicated that the nursing population has positive perception on the safety of patients. Table 2 shows that 69% of nurses rated their units as either excellent or very good in relation to patient safety, with very few (1.8%) rating them as poor or failing. Almost 96% of MSW nurses rated patient safety in their hospital as being above the acceptable level. This indicates that hospital management/managers maintain an organizational climate that ensures patient safety in the MSW and that nurses understand that patient safety should be given top priority.

### 3.3. Number of Adverse Incidents Reported

This section of the survey provides information on the number of incidents reported by each nurse in the past year based on their subjective accounts. Table 3 shows 50% of RNs from all four hospitals had not reported any safety incident in the past year, while a third of nurses reported one to two events. Perhaps the low incident rate could be due to unnoticed events or people failing to report them. The mode score of the number of incidents reported in the past year in the MSW was one. 

### 3.4. Perceived Culture of Their Ward

**Figure 2:**
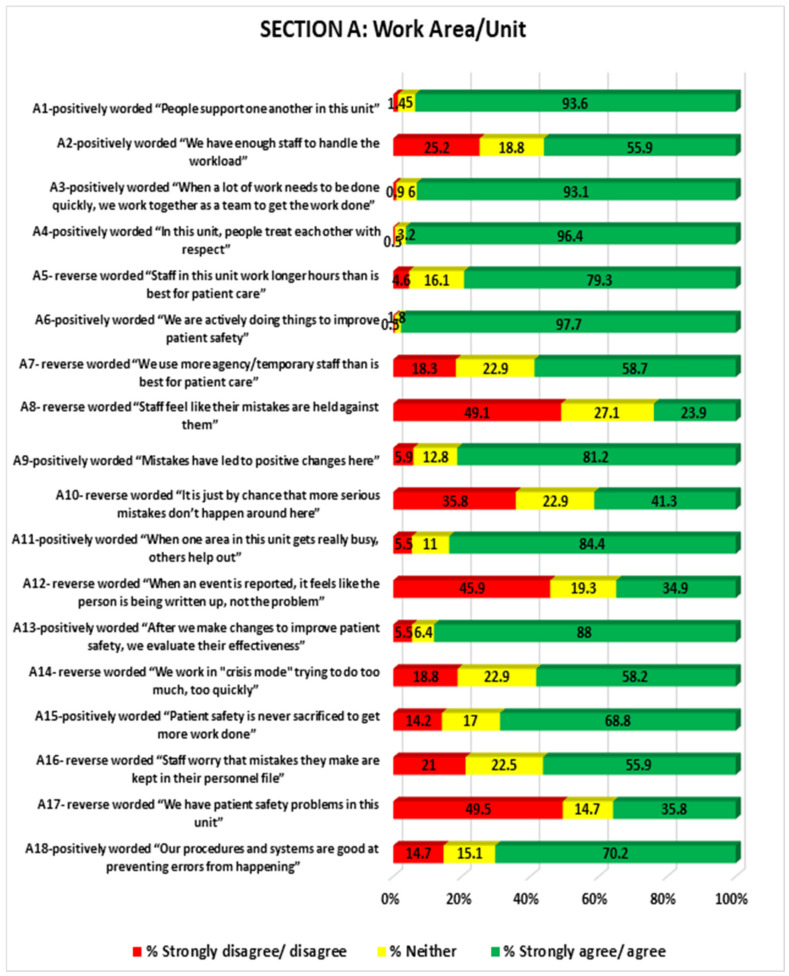
Showing work area/unit patient safety.

This section of the survey reports on participants’ perceptions of the culture within the wards of the four hospitals. The findings suggest that the majority of respondents agreed that they supported each other in sharing their responsibilities in order to ensure a culture of patient safety, which infers that there could be a tendency for mutual aid or support and unanimity over their perception towards safety culture. This attitude of support suggests that good teamwork and a positive view of the importance of patient safety prevails among the facilities (Figure 2).

The hospitals were found to have adequate RNs who valued mutual respect in the workplace and knew their job responsibilities, could manage caseloads, and could provide the best patient care so as to ensure patient safety. One way to ensure this would be to hire an agency of staff, and when nurses are found to not admit to their mistakes, they would be brought before the agency. If no adverse incidents are reported in the wards, then the effectiveness of the patient safety improvements is evaluated by considering, for example, whether staff are helpful during busy times and whether nurses can work quickly to complete a job, making sure there is no sacrifice in terms of patient safety. However, this could result in staff worrying about mistakes being kept in their files. Overall, this could result in good procedures for the prevention of medication errors. 

Additionally, this section has provided valuable, albeit at times conflicting, information regarding nurses’ perception of reporting and blame relating to errors, as well as their perceptions of teamwork. There are other significant issues that stem from nurses working long hours, which, because of fatigue, might affect their ability to safely administer medication. A considerable number of nurses spoke of concerns that any mistakes would be held against them.

However, in this study, 35% of nurses disagreed that event reporting is an issue. There was also a difference in concern between medical units and surgical units—those in medical units showed less concern about the repercussions of reporting medical errors than those in surgical units. It appears this concern decreased based on the size of the hospital. There was less concern in the large hospital (F), and more concern in hospitals B, S, and A. 

### 3.5. Nurses’ Perceptions of Their Supervisor/Manager

Section 5 measured the relationship between the RN and supervisors/managers in everyday routines in the MSW. The majority (89.9%) of nurses agreed their supervisors/managers made affirmative comments when nurses followed professionally established procedures upon carrying out their work. The majority of nurses (87.6%) also said their supervisors and managers genuinely cared about their suggestions regarding patient safety. However, 48.2% of nurses said their supervisors/managers at times put pressure on them to finish the work quickly. This tendency was similar among all four hospitals, irrespective of the medical or surgical wards. 

## 4. Discussion

The survey showed that overall, the results indicated that the participating nursing population had a positive perception about the safety of patients. This is consistent with existing literature and concludes that the nurses’ opinions on patient safety culture were influenced by many aspects, including nurse-specific factors such as competency, age, education level, and external factors or environment [7]. The external factors were leadership, hospital policy, teamwork, management support, communication openness, promotion, and reward [8]. In this study, the five leading positive perceptions among nurses regarding patient safety cultures in the working place were as follows: actively doing things to improve patient safety, healthcare team members respecting and supporting each other, teamwork to finish work on difficult timelines, and changes in patient safety to ensure methods were obeyed, followed by an appraisal system to assess their effectiveness. Moreover, roughly half of the nurses disagreed with negative perceptions, such as safety problems in their hospitals, healthcare professionals feeling mistakes were held against them, and adverse safety incidents being reported in way to target the involved professional. This overall positive perception is similar to those reported in previous studies among nurses. Thomas-Hawkins and Flynn, 2015, concluded that the majority of nurses working in dialysis units rated their overall safety practices to be good to excellent [4]. Similarly, if we compare the results at the Saudi Arabian healthcare facilities, Alahmadi, 2010, reported that a majority of the health professionals including nurses rated patient safety culture to be very good to excellent [9]. Taken together, out results corroborate previous findings about positive perceptions regarding patient safety among nurses [4,9]. 

Medical errors are routinely encountered by nursing staff and personal; subjective perceptions of errors are associated with patient safety culture [10]. Meaningful insight into the enactment of a safety culture will produce significant outcomes. This is beneficial to nurses, nurse managers, and hospital management [11].

A Saudi Arabian study of hospitals in Riyadh reported several hospitals lacked core practices for improving patient safety [9]. Reasons posted were education, training, quality management, data usage, non-rewarding environment, and the quality of strategic planning [12]. Hospitals should emphasize frankness, communication, teamwork, and effective leadership to improve patient safety, as these are needed to provide integrated care [13]. However, it is noteworthy that half of the study participants neither completed nor submitted a report related to medical errors in the past year. Furthermore, half of study participants expressed concerns about being accused of a medical error. Only two out of the four hospitals in which the study was carried out could produce statistics related to the reported medication errors, whereas the other two hospitals had no data on medical errors. 

Similar to the literature, this study found many barriers to reporting medication errors by nurses. Using the Yorkshire Contributory Factors Framework (YCFF) [14], the barriers found could be grouped into four levels. First, active failure and circumstantial factors included human nature and a lack of feedback mechanisms. Second, conditions that pertained to local workplaces included caseload, inadequacy of nursing faculties and nursing leadership, and blame in the workplace. Third, hidden organizational and external factors such as insufficient knowledge and skills, as well as lack of precision or noncompliance with policies, were other barriers. Fourth was fear of reporting an error. These barriers need to be addressed by nursing management teams and quality offices to encourage error reporting and to improve patient safety. Teamwork between and/or within units, and continuous improvement through learning, overall perceptions of patient safety, transitions, communication openness, and less incidents of reported events were considered to be strengths. No-punitive response to errors were ascertained as weaknesses in the patient safety culture [15]. Introducing clinical governance, supplying appropriate workforce, providing nurses with education on patient safety, and disseminating sound communication processes were pinpointed as approaches to improve safety culture in hospitals [16].

## 5. Implications

Patient safety measures rely on the perception of individuals, leadership, and the organizational structure. This study adds to the findings of the existing literature, especially through evidential proof to the Yorkshire Classification. Although the overall perception of nurses toward patient safety is excellent, the fact that only a small number of incidents were reported by RNs could be due to a fear of punishment. To overcome this, hospitals should establish specific strategies to improve the safety culture. However, that the fact that the sample size was not estimated based on the power analysis is a weakness. It may not be out of place to mention that the final sample size was comparable to some of the previous studies on similar aspects. Alahmadi, 2010, reported findings about patient safety cultures based on a sample of 223 health professionals [9]. A recent systematic review summarized that healthcare professionals are usually reluctant about recording and reporting patient safety incidents [17]. Therefore, it is necessary to bear in mind about desirability bias in the self-reported accounts of patient safety-related measures.

## 6. Recommendations

This study can have a profound effect on RNs, supervisors/managers, hospital administrators, and health care systems for advancing patient safety and reducing medical errors, by employing both reactive and proactive strategies. Proactive strategies prospectively identify the weak points of the organization and address them. Reactive strategies are learning from reported incidents. To facilitate this, the aim should be a culture of safety instead of blame or shame. With an intimidation-free open channel of communication, a burnout-free working schedule, supportive leadership, and adequate training, barriers to reporting medical errors can be broken.

## 7. Conclusions

In conclusion, 69% of RNs rating their wards as having great/excellent safety culture. Moreover, some participants felt it was problematic that blame was assigned to nurses for reported errors. While 55.9% of participants noted that all errors or narrowly avoided errors had been reported, less than half actually reported errors in the last year. Finally, the perceived safety culture was largely positive; however, the results also indicated that a culture of safety comes with some risk and blame.

## Figures and Tables

**Table 1:** Response workplace and units.

Name of the Hospital	Medical Unit	Surgical Unit	Total	Percent
Hospital F	31	30	61	28.0
Hospital B	24	28	52	23.9
Hospital S	34	40	74	33.9
Hospital A	14	17	31	14.2
Total	103	115	218	100.0

**Table 2:** Patient safety grade.

Patient Safety Grade	Frequency	Percent
Excellent	67	30.7
Very good	83	38.1
Acceptable	59	27.1
Poor	4	1.8
Failing	5	2.3
Total	218	100.0

**Table 3:** Number of events reported.

Number of Incidents Reported in the Past Year	Frequency	Percent
No event reports	109	50.0
1–2 event reports	69	31.7
3–5 event reports	32	14.7
6–10 event reports	8	3.7
Total	218	100

## Data Availability

Data from questionnaires were kept in a locked filing cabinet and a password secured electronic folder accessible only by the analyst. The data will be kept for up to 5 years after publishing and all reports will then be destroyed or erased as appropriate.
